# Doxorubicin–Mediated miR–433 Expression on Exosomes Promotes Bystander Senescence in Multiple Myeloma Cells in a DDR–Independent Manner

**DOI:** 10.3390/ijms24076862

**Published:** 2023-04-06

**Authors:** Elisabetta Vulpis, Lorenzo Cuollo, Cristiana Borrelli, Fabrizio Antonangeli, Laura Masuelli, Marco Cippitelli, Cinzia Fionda, Giulio Caracciolo, Maria Teresa Petrucci, Angela Santoni, Alessandra Zingoni, Alessandra Soriani

**Affiliations:** 1 Department of Molecular Medicine, Sapienza University of Rome, Laboratory Affiliated to Istituto Pasteur Italia—Fondazione Cenci Bolognetti, Viale Regina Elena 291, 00161 Rome, Italy; 2 Institute of Molecular Biology and Pathology, National Research Council (CNR), 00161 Rome, Italy; 3 Department of Experimental Medicine, Sapienza University of Rome, 00161 Rome, Italy; 4 Department of Molecular Medicine, Sapienza University of Rome, 00161 Rome, Italy; 5 Department of Cellular Biotechnologies and Hematology, Sapienza University of Rome, 00161 Rome, Italy; 6 IRCCS Neuromed, 86077 Pozzilli, Italy

**Keywords:** multiple myeloma, senescence, miRNAs, exosomes

## Abstract

The success of senescence-based anticancer therapies relies on their anti-proliferative power and on their ability to trigger anti-tumor immune responses. Indeed, genotoxic drug-induced senescence increases the expression of NK cell-activating ligands on multiple myeloma (MM) cells, boosting NK cell recognition and effector functions. Senescent cells undergo morphological change and context-dependent functional diversification, acquiring the ability to secrete a vast pool of molecules termed the senescence-associated secretory phenotype (SASP), which affects neighboring cells. Recently, exosomes have been recognized as SASP factors, contributing to modulating a variety of cell functions. In particular, evidence suggests a key role for exosomal microRNAs in influencing many hallmarks of cancer. Herein, we demonstrate that doxorubicin treatment of MM cells leads to the enrichment of miR-433 into exosomes, which in turn induces bystander senescence. Our analysis reveals that the establishment of the senescent phenotype on neighboring MM cells is p53- and p21-independent and is related to CDK-6 down-regulation. Notably, miR-433-dependent senescence does not induce the up-regulation of activating ligands on MM cells. Altogether, our findings highlight the possibility of miR-433-enriched exosomes to reinforce doxorubicin-mediated cellular senescence.

## 1. Introduction

Multiple myeloma (MM) is regarded as the second most common hematological malignancy, accounting for approximately 1% of all neoplastic diseases [1]. MM is characterized by the abnormal proliferation of malignant plasma cells (PCs) within the bone marrow (BM), which entails bone lesions, anemia, hypercalcemia and the abnormal production of monoclonal immunoglobulins, resulting in kidney damage. The peculiar microenvironment created by MM in the BM not only supports the proliferation of malignant PCs, increasing their resistance to chemotherapeutic agents, but it also proved to be highly immunosuppressive [2].

We have previously demonstrated how sub-lethal doses of genotoxic drugs such as doxorubicin (DOX) and melphalan (MEL) are able to induce an immunogenic type of senescence in MM cells. Indeed, senescent MM cells, in addition to arresting their proliferation, also increase the expression of NK cell-activating ligands, thus stimulating degranulation and IFN-γ release by NK cells [3,4,5,6]. On the other hand, senescent cells are typically characterized by the so-called senescence-associated secretory phenotype (SASP), which implicates the secretion of large amounts of soluble factors, such as inflammatory cytokines, growth factors and matrix-degrading enzymes, which have been associated with tumor-promoting and immunomodulatory effects [7]. The SASP is also responsible for the induction of DNA-damage response (DDR) and senescence in bystander cells that never experienced direct damage, a phenomenon best known in the context of irradiation, which appears to be driven by oxidative stress [8,9]. 

Recently, extracellular vesicles (EVs), including exosomes, have been considered as SASP factors since they are abundantly secreted by senescent cells and contribute to modulating several functions in recipient cells [10,11,12]. EVs are recognized as important mediators of intercellular communication due to their ability to carry and transfer a wide variety of molecules, such as microRNAs, messenger RNAs, DNA, lipids and proteins. EVs can exert paracrine effects in BM and can be found in the bloodstream as well; some reports suggest a role of EVs in MM growth and dissemination [13,14,15] in contributing to bone disease [16].

In this work, we found that exosomes released by DOX-induced senescent MM cells carry miR-433; moreover, miR-433-enriched exosomes induce elevated senescence-associated β-galactosidase (SA-β-gal) activity, a marker of cellular senescence, in MM cells in the absence of DDR activation. In particular, we did not observe increased levels of p21 and p53 phosphorylation nor the up-regulation of NK cell-activating ligands. Altogether, we demonstrated that miR-433-enriched exosomes might affect the MM tumor microenvironment, leading to a state of senescence-associated cell-cycle arrest, not necessarily irreversible, reinforcing and spreading senescence initiated by the primary DOX treatment of MM cells.

## 2. Results

### 2.1. Exosomes Released by DOX-Treated Multiple Myeloma Cells Induce a Senescence-Like Phenotype on Bystander Cells 

**Figure 1:**
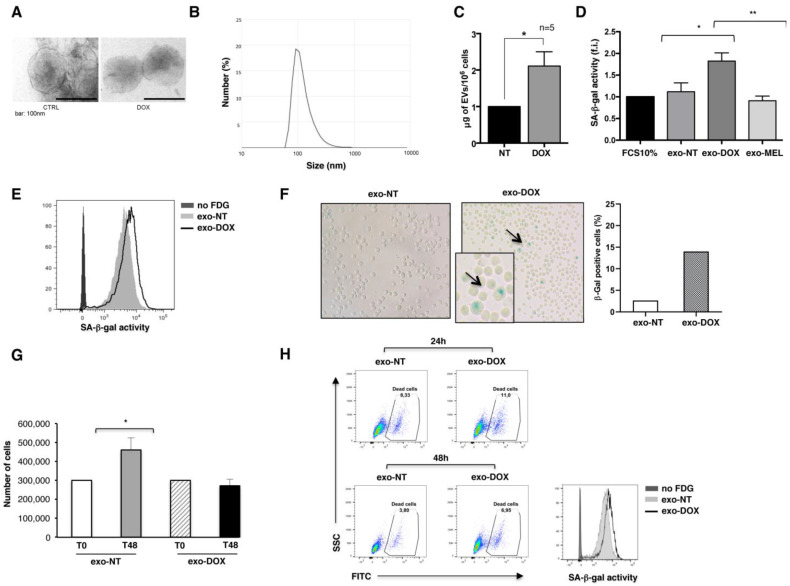
DOX-treated SKO-007(J3) multiple myeloma cells release exosomes able to induce a senescence phenotype. (**A**) SKO-007(J3) cells were incubated with doxorubicin (DOX) (0.05 μM) for 48 h and left for an additional 24 h in the absence of the drug. Exosomes were then isolated from the supernatants and characterized by transmission electron microscopy. A representative picture of SKO-007(J3)-derived exosomes is shown. Scale bar, 100 nm. (**B**) A representative experiment of exosome size distribution by Dynamic Light Scattering (DLS) m (nm scale) is shown. (**C**) Exosomes released by untreated and DOX-treated SKO-007(J3) were quantified by Bradford assay, normalized for 1 million cells and plotted as fold increase between untreated and DOX-treated cells. The mean of five independent experiments is shown. (**D**) SKO-007(J3) cells were incubated with exosomes (20 μg/mL) derived from untreated, DOX-treated and MEL-treated SKO-007(J3) cells. After 5 days, cells were washed and then incubated 1 h with bafilomycin A1 (100 nM) to induce lysosomal alkalinization, followed by 1 h incubation with C12FDG (33 μM). SA-β-gal^high^ senescent cells were analyzed by flow cytometry. Data are expressed as a fold increase in respect to untreated control cells and are the result of five different experiments. (**E**) MM cells were treated with a higher number of exosomes (50 μg/mL) for 24 h and analyzed by flow cytometry for the presence of senescent cells. A representative experiment is shown. (**F**) In parallel, SKO-007(J3) cells were fixed and incubated overnight at 37 °C without CO_2_ with SA-β-gal stain solution (“Senescence-associated β-galactosidase staining”). Senescent cells were identified as blue-stained cells and counted using microscopy. Magnification 200×. Results are representative of one of two independent experiments. (**G**) SKO-007(J3) cells were left untreated (T0) or treated for 48 h (T48) with exo-NT and exo-DOX, live cells were then counted by Trypan Blue staining using light microscopy. (**H**) SKO-007(J3) cells were treated with exo-NT and exo-DOX (50 μg/mL) for 24 and 48 h; the percentage of dead cells was measured by flow cytometry using Zombie Green. SA-β-gal activity was measured at 48 h to confirm the induction of senescence in the same experiment. A representative experiment is shown. Statistical analysis was performed with Student’s *t*-test (* *p* < 0.05, unpaired *t*-test, ** *p* < 0.01, unpaired *t*-test).

Exosomes are now recognized as important components of senescent cell’s secretome; still, their role in the phenomenon of bystander senescence remains largely unexplored. Several studies have shown that stress conditions as well as chemotherapeutic treatments can stimulate exosome secretion from cancer cells. For this reason, we isolated exosomes from the conditioned media of untreated or drug-exposed SKO-007(J3) MM cells as previously reported [17] and characterized them by transmission electron microscopy showing the presence of nano-sized cup shaped vesicles (Figure 1A). Furthermore, we employed dynamic light scattering (DLS) methodology to estimate vesicle size distribution, measuring an average diameter of 96 ± 8 nm (Figure 1B). Accordingly, as previously demonstrated upon MEL treatment [17] we measured increased amounts of exosomes from DOX-treated MM cells (Figure 1C). 

Subsequently, we endeavored to address whether MM-derived exosomes have the capability to induce a bystander senescent phenotype. To this purpose, we measured SA-β-gal activity, the most typical marker of cellular senescence. MM cells were incubated for five days with 20 μg/mL of exosomes derived from untreated, MEL- and DOX-treated MM cells (exo-NT, exo-MEL and exo-DOX), and the SA-β-gal activity was measured by flow cytometric analysis, as indicated by the increased MFI (mean fluorescence intensity). As shown in Figure 1D, MM cells exposed to DOX-derived exosomes showed enhanced SA-β-gal activity, similarly to the MM cells directly exposed to DOX genotoxic drug [4], whereas we did not observe any effect in response to MEL-derived exosomes. To strengthen these data, a larger number of exosomes (50 μg/mL), isolated from untreated and DOX-treated cells, were incubated for 24 h with MM cells, and we found that MM cells displayed the characteristic of cellular senescence more rapidly, namely an enlarged morphology and increased percentage of SA-β-gal-positive cells, as revealed both by FACS analysis (Figure 1E) and by microscopy through the quantification of SA-β-gal-positive cells, which show the typical presence of a blue precipitate in the cytoplasm (Figure 1F). Accordingly, a proliferation arrest was observed in MM cells treated with DOX-derived exosomes, as shown by the reduction in cell number after 48 h of treatment (Figure 1G). Such an increase in SA-β-gal activity induced by exo-DOX was not paralleled by a significant difference in cell death, as measured by flow cytometry with live/dead staining (Figure 1H).

Since treatment-induced senescence on MM cells is known to be accompanied by the up-regulation of NK cell activating ligands [4], we then investigated the ability of the exosomes derived from senescent MM cells to boost NK-cell-mediated killing. The expression of the degranulation lysosomal marker CD107a was evaluated by immunofluorescence and flow cytometric analysis by gating on primary NK cells upon their interaction with MM cells, previously incubated for 24 h with exo-NT and/or exo-DOX. The levels of NKG2D and DNAM-1 ligands, involved in NK cell-mediated cytotoxicity, were similar in both exo-treated samples (Figure S1A). As shown in Figure S1B,C, the expression of CD107a on NK cells contacting exo-DOX-treated senescent MM target cells indicates that those cells are not more susceptible to NK cell lysis compared to cells treated with exo-NT. 

### 2.2. Exosomes Released by DOX-Treated MM Cells Convey miR-433

As evidence in the literature suggests that exosomes may contain and store doxorubicin [18], we analyzed the possible direct role of the drug in the senescence-like phenotype on bystander cells. To this end, the cells were deprived of treatment with DOX 24 h before exosome extraction, and using flow cytometry, we evaluated whether exosomes, recognized by the expression of the specific marker CD63, showed fluorescence emission within the phycoerythrin (PE) channel, which detects the intrinsic fluorescence of DOX with high sensitivity. The comparable levels of autofluorescence between exo-DOX and exo-NT demonstrate that these vesicles do not carry a measurable amount of the drug in our experimental setting (Figure S2). Therefore, exosomes convey other bioactive molecules that are responsible for the induction of bystander senescence. 

**Figure 2:**
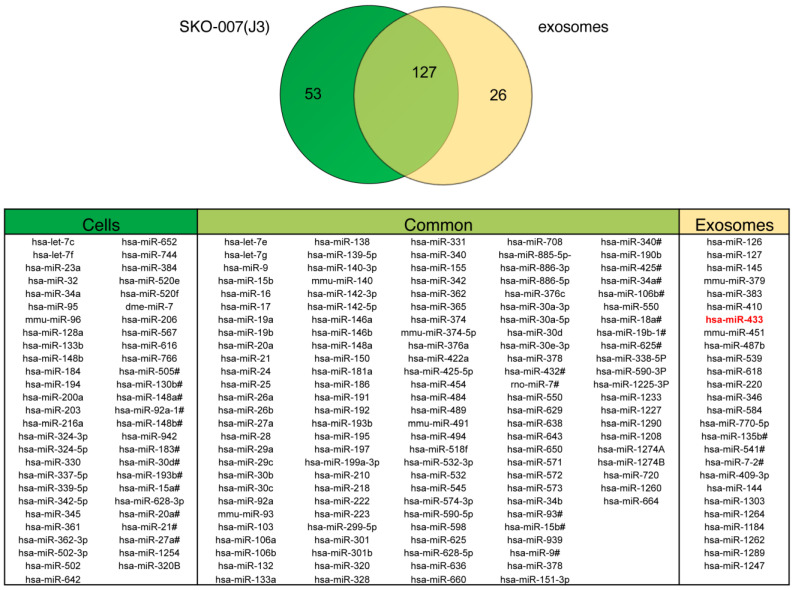
miRNA profiling of MM cell-derived exosomes and parental SKO-007(J3) cells. Total RNA was isolated from both SKO–007(J3) cells and SKO-007(J3)-derived exosomes and analyzed for microRNA expression profiling using Megaplex Pool cards A and B, as described in Materials and Methods. The Venn diagram was created using an online program (http://www.bioinformatics.lu/venn.php, accessed on 1 April 2023), and the data are reported in the table. In the left column of the table, the miRNAs expressed only in SKO-007(J3) cells are shown, the central column displays the miRNAs present in both SKO-007(J3) cells and SKO-007(J3)-derived exosomes and the right column shows those expressed only in SKO-007(J3)-derived exosomes. The results of the representative experiments are shown.

**Figure 3:**
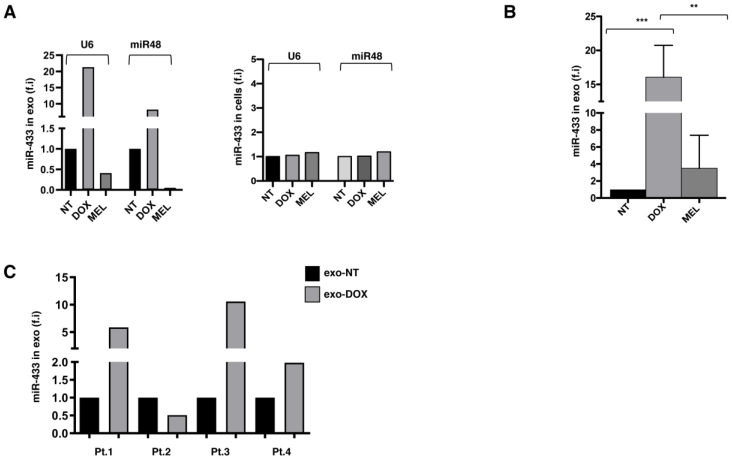
Exosomes derived from DOX-treated MM cells carry miR-433. (**A**) SKO-007(J3) cells were left untreated or treated with DOX and MEL, as described above. The expression of mir-433 was evaluated on both SKO-007(J3)-derived exosomes and SKO-007(J3) cells by real-time PCR. U6 and miR-48 were used to normalize miR-433 expression. (**B**) SKO-007(J3) cells were treated as described above and the expression level of miR-433 on the exosomes was verified by real-time PCR. The average of four different experiments is shown. Statistical analysis was performed using Student’s *t*-test (** *p* < 0.01, unpaired *t*-test, *** *p* < 0.001, unpaired *t*-test). (**C**) Bone marrow mononuclear cells (BMMCs) derived from 4 MM patients were left untreated or treated with DOX (0.05 μM) for 48 h. RNA was extracted from the exosomes (isolated from supernatants) and analyzed for the expression of miR-433 by real-time PCR (normalized with U6).

Since exosomes are reportedly an important source of miRNAs, real-time qPCR was performed on total RNA extracted from both exosomes and cells to generate a miRNA expression profile aimed at identifying exosome-carried miRNAs potentially involved in the senescence phenotype. Among the different miRNAs selectively enriched in exosomes, we focused our attention on miR-433 (Figure 2), which has been previously described to contribute to the induction of senescence in ovarian cancer cells by down-modulating the expression of the cyclin-dependent kinase CDK6 [19]. Notably, miR-433-3p presents a motif in the 3′ half (UCCU) associated with the active loading of miRNAs into exosomes [20]. Thus, we investigated whether the drug treatment of MM cells could enhance the loading of miR-433 into exosomes. We observed the exclusive presence of miR-433 in the exosomal cargo in respect to parental cells (Figure 3A) with a substantial increase in miR-433 abundance in exo-DOX compared to exo-NT and exo-MEL (Figure 3B). Importantly, the DOX treatment of bone marrow mononuclear cells (BMMCs) derived from MM patients (Table 1) increased the amount of miR-433 loaded in exosomes isolated from the conditioned medium (Figure 3C), suggesting that our observation is not restricted to the SKO-007(J3) cell line.

### 2.3. Overexpression of miR-433 in MM Cells Results in the Induction of a Senescent Phenotype through CDK6 Down-Modulation

**Figure 4:**
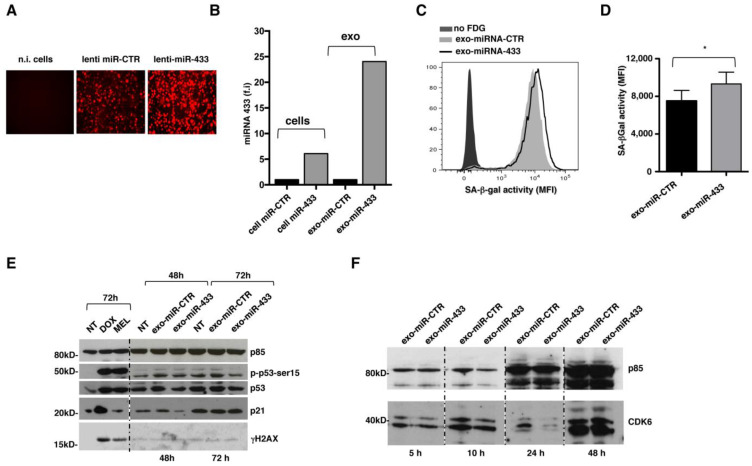
Exosomal transfer of miR-433 can promote a senescent phenotype through CDK6 down-modulation on MM cells. (**A**) Lentiviral-vectors overexpressing miR-433 or miR-CTR fused in-frame with mCherry were used to infect SKO-007(J3) cells. Cells were washed with PBS and visualized by fluorescence microscopy to determine the presence of mCherry-positive cells. (**B**) The expression of mir-433 was evaluated on infected SKO-007(J3) cells and their derived exosomes by real-time PCR 6 days post-infection. A representative experiment is shown. (**C**) Exosomes isolated from SKO-007(J3) cells infected with the lentiviral constructs were incubated with MM cells; SA-β-gal activity on MM cells was measured using flow cytometry upon 48 h of incubation. (**D**) The average of three different experiments is shown (incubation time of 6 days). Statistical analysis was performed with Student’s *t*-test (* *p* < 0.05, unpaired *t*-test). (**E**) Western blot analysis of p53, phospho-p53 (Ser15), p21 and γH2AX in SKO-007(J3) cells treated with exo-miR-CTR and exo-miR-433 for 48 and 72 h. p85 was used as protein loading control. A representative Western blot is shown. Irrelevant lanes were removed between lanes (dotted line). (**F**) SKO-007(J3) cells were treated with exo-miR-CTR and exo-miR-433 for the indicated times. Cell lysates were immunoblotted with anti-CDK6 or with anti-p85, used as loading control. A representative Western blot is shown. Irrelevant lanes were removed between lanes (dotted line).

To better examine the functional consequence of the exosomal expression of miR-433, a lentiviral vector overexpressing the precursor of miR-433 was used together with a matched miR-control (miR-CTR). The stable integration of the construct was confirmed by fluorescence microscopy, monitoring the red signal from red fluorescent protein (RFP) in infected MM cells (Figure 4A), and by real-time PCR analysis (Figure 4B). A marked enrichment of miR-433 was observed in exosomes isolated from cells infected with the lentivirus carrying the miR-433 precursor (exo-miR-433), again confirming the specific loading of miR-433 into exosomes. As control, we assessed the expression of miR15b, which predictably maintains the same expression levels in cells expressing miR-433 or the matched miR-CTR. 

As shown in Figure 4C,D, exosomes derived from miR-433 infected cells were able to trigger a senescent phenotype upon 24 h of incubation without triggering DDR, since the protein levels of p53 and p21, as well as p53 phosphorylation on Ser15 and histone H2AX phosphorylation (γH2AX), were comparable between exo-miR-CTR- and exo-miR-433-stimulated cells (Figure 4E). As a control, we stimulated cells with DOX and MEL, which, as already described [21], induced DDR activation. The densitometric analysis is reported in Table S1.

Notably, the kinetics of CDK6 expression by MM cells showed a marked down-regulation upon 24 h exposure to exo-miR-433, and the subsequent increase to the initial level after a further 24 h of incubation (Figure 4F) suggested that the observed phenotype may be due to the reversible down-modulation of CDK6, which results in a reduced capacity to phosphorylate Rb, hindering progression through the cell cycle. 

Collectively, these data highlight that DOX-treated MM cell-derived exosomes can induce a senescent-like phenotype, independent of DDR, through miR-433-driven CDK-6 down-regulation. 

## 3. Discussion

Senescent cells show vast changes in gene expression, which underlie the increased secretion of many types of soluble factors, a phenomenon collectively referred to as senescence-associated secretory phenotype (SASP). The secretome of the senescent cell is composed of several cytokines, chemokines, growth factors and proteases [9], which overall can have a profound effect on the microenvironment, promoting or repressing tumor progression in a context-dependent manner [5,22,23]. The pro-inflammatory role of SASP, resulting in the enhanced proliferation and tumorigenesis of epithelial cells, the promotion of angiogenesis, the promotion of cancer cell invasiveness and the increased growth of xenograft tumors in vivo, has been well described in several works [24]. On the other hand, the involvement of many SASP components in the clearance of senescent hepatic stellate cells in vivo [25] and, more recently, in the activation and recruitment of anti-tumor immune cells, has also been reported [5,23]. Moreover, SASP factors might contribute to tumor suppression by reinforcing autocrine senescence or inducing senescence in paracrine manner.

Extracellular vesicles, initially described as cell debris, are now recognized as important mediators of intercellular communication, due to their ability to transport a wide array of molecules, such as cytokines, inflammatory mediators and miRNAs [26,27]. We and other groups demonstrated that exosome release increases during cellular senescence [17,28,29], and it is now generally accepted that exosomes constitute part of the SASP themselves. However, the effects of senescence on exosomes biogenesis and secretion, as well as the role of exosomes in cellular senescence are still matter of debate. Several studies performed in different tumor models suggest a role for miRNAs in cellular senescence [30,31]. 

Our data showed the presence of miR-433 in exosomes isolated from SKO-007(J3) human MM cells, which is substantially enriched upon sub-lethal doxorubicin treatment. Doxorubicin is a commonly used frontline chemotherapeutic agent for cancer, and it is known to induce senescence by a p53-dependent pathway in tumor cells, including MM [32]. miR-433 possesses a sorting sequence (the motif UCCU) that determines its loading in exosomes [20,33]. This finding strengthens the hypothesis that exosomal miR-433 could inhibit target genes in the paracrine way, opening the opportunity of a therapeutic targeting of microRNAs in the tumor modulating miRNA retention or secretion through exosomes [20]. Indeed, miR-433 has been previously associated with the ability to induce senescence in neighboring cells upon overexpression in ovarian cancer cells [19]. 

Several independent studies demonstrated the anti-tumor role of miR-433 [34,35,36,37]. Interestingly, it has been recently demonstrated that circ_0003489, highly expressed in MM tissue, enhanced MM progression by sponging miR-433-3p [37]. 

Our data suggest that miR-433 can induce senescence independently from the canonical senescence program mediated by p53 and p21 and, more importantly, can sustain and amplify senescence initiated through the doxorubicin treatment of MM cells. The long-term implications of senescence induction, potentially detrimental, need to be considered. At the same time, SASP-related chronic inflammation and cell-cycle re-entry of stemness-reprogrammed senescent cancer cells must be avoided. Thus, a deeper knowledge of the SASP induced by exo-miR-433 in our model is needed. 

The findings of this work expand the current knowledge on exosomes-mediated senescence and provide a novel understanding of the relationship between miR-433 and senescence, shedding light on the impact that anti-tumor therapy has on the secretion and function of these vesicles in a model of MM. 

## 4. Materials and Methods

### 4.1. Cell Lines and Clinical Samples 

The SKO-007(J3) MM cell line was provided by Prof. P. Trivedi (Sapienza University of Rome, Italy). After thawing, cells were maintained in culture for no longer than four weeks and tested periodically for mycoplasma contamination. The SKO-007(J3) cell line was authenticated by IRCCS Azienda Ospedaliera Universitaria San Martino-IST, S.S. Banca Biologica e Cell factory. Bone marrow samples from multiple myeloma patients were managed at the Department of Cellular Biotechnologies and Hematology, Institute of Hematology (“Sapienza” University of Rome, Italy). The study was conducted in accordance with the Declaration of Helsinki and approved by Ethics Committee of the “Sapienza” University of Rome (Rif. 5191).

### 4.2. Treatments

SKO-007(J3) MM cell line was treated with sub-lethal doses of doxorubicin (DOX) and melphalan (MEL), established by MTT assay as previously described [4] for 48 h, at a density of 3 × 10^5^/mL; afterwards, cells were washed twice to eliminate residual drugs and left for a further 24 h with an absence of drugs. 

### 4.3. Senescence Associated-β-Galactosidase Staining

For microscopy, cells were spotted on a poly-L-lysine-coated glass slide and spun (cytospin) at 700 RPM for 3 min. Subsequently, cells were fixed with 2% formaldehyde for 3 min, washed in PBS and left overnight at 37 °C without CO_2_ in the presence of fresh SA-β-Gal staining solution. The composition of the staining solution was the following: 1 mg/mL 5-bromo-4-chloro-3-indolyl β-D-galactopyranoside (X-Gal), 150 mM NaCl, 2 mM MgCl_2_, 40 mM citric acid/sodium phosphate buffer (pH 6.0), 5 mM potassium ferrocyanide and 5 mM potassium ferricyanide. Cells showing the characteristic blue precipitate in the cytoplasm, identified and counted by standard light microscopy, were recognized as senescent. Images were acquired using an EVOS microscope, magnification 200×. 

For flow the cytometric determination of cellular senescence, SA-β-Gal activity was evaluated using the substrate C12FDG (Invitrogen), as reported in the literature [38].

### 4.4. Immunofluorescence and Flow Cytometry

Unconjugated monoclonal antibodies used for immunostaining were the following: anti-MICA (MAB159227) and anti-MICB (MAB236511) from R&D Systems (Minneapolis, MN, USA) and anti-PVR (46.31 or SKII.4), which was kindly provided by Prof. Colonna (Washington University in St. Louis, MO, USA). Allophycocyanin (APC)-conjugated goat affinity purified F(ab′)2 fragment to mouse IgG (GAM) was purchased from Jackson ImmunoResearch Laboratories (West Grove, PA, USA). 

Dead cells were quantified with Zombie Green Fixable Viability Kit (BioLegends). All acquisitions were performed on a FACSCanto II (BD Biosciences, San Jose, CA, USA). Flow cytometric analysis was performed using the FlowJo software version 8.8.7 (TreeStar, Ashland, OR, USA). 

### 4.5. Exosome Isolation 

Exosome-free medium was obtained by centrifugating fetal calf serum (FCS) at 100,000× *g* for 3 h in a Beckman ultracentrifuge (Beckman Coulter, Brea, CA, USA). RPMI 1640 was supplemented with 10% of exosome free-serum and antibiotics. SKO-007(J3) MM cell line was cultured at 0.8–1 × 10^6^ cells/mL in exosome-free medium for 48 h. Exosome purification was performed by sequential centrifugations, as previously reported [5]. Cells were eliminated by centrifugation at 300× *g* for 10 min and supernatants were collected. The supernatants were subsequently centrifuged at 2000× *g* for 20 min, followed by a second centrifugation at 10,000× *g* for 30 min in order to remove debris. The supernatants obtained were finally filtered (0.22 μm filter) and ultracentrifuged at 100,000× *g* for 70 min at 4 °C to isolate exosomes, which were first washed with cold PBS and then centrifuged a second time at 100,000× *g* for 70 min at 4 °C. At the end of the procedure, the exosomes were resuspended in 100–250 μL of PBS for further experiments. 

### 4.6. Transmission Electron Microscopy

Transmission electron microscopy was performed as already reported [17]. Exosomes were fixed in 2% paraformaldehyde and adsorbed on formvar-carbon-coated copper grids, which were then incubated in 1% glutaraldehyde for 5 min, washed thoroughly with deionized water and then negatively stained with 2% uranyl oxalate (pH 7.0) for 5 min and methyl cellulose/uranyl for 10 min at 4 °C. The excess methyl cellulose/uranyl was eliminated by blotting, and the grids were finally air-dried and analyzed with a transmission electron microscope (Philips Morgagni268D) at an accelerating voltage of 80 kV. Digital images were obtained using Mega View imaging software.

### 4.7. Size Experiments through Dynamic Light Scattering (DLS)

Dynamic Light Scattering (DLS) experiments were performed in order to measure exosome size distribution [39]. All the measurements were conducted at 25 °C on a Zetasizer Nano ZS90 spectrometer (Malvern, UK) equipped with a 5 mW HeNe laser (wavelength λ D 632.8 nm) and a non-invasive back-scattering optical setup (NIBS). For each sample, the detected intensity was processed by a digital logarithmic correlator, which computes a normalized intensity autocorrelation function. Then, the distribution of the diffusion coefficient D was obtained by using the CONTIN method.63 D was converted into an effective hydrodynamic diameter DH through the Stokes-Einstein equation: DH D KBT/(3phD), where KBT is the system’s thermal energy and h represents the solvent viscosity. Solvent-resistant micro cuvettes (ZEN0040, Malvern, Herrenberg, Germany) have been used for experiments with a sample volume of 40 μL. 

### 4.8. Analysis of Exosomes by Flow Cytometry 

To analyze exosomes by flow cytometry, exosomes were captured using CD63+ magnetic dynabeads (Invitrogen). Briefly, 10 μg of exosomes were diluted in PBS 0.1% Bovine Serum Albumin (BSA) (final volume 100 µL), and then incubated with 20 µL of anti-CD63 antibodies-conjugated magnetic beads, for 18–22 h at 4 °C with gentle rotation. The bead-exosome conjugates were finally resuspended in 200 µL of buffer and stained with control IgG/PE or anti-CD63/PE (BioLegend). Flow cytometric analysis was performed on a FACSCantoII (BD Biosciences, San Jose, CA, USA); data were analyzed and plotted using the FlowJo software version 8.8.7.

### 4.9. Exosomal RNA Isolation

Exosomal RNA was purified from 100 μg of exosomes using a Total Exosome RNA and Protein Isolation Kit (Invitrogen), according to the manufacturer’s protocol. Exosomes, resuspended in PBS, were first diluted in PBS (final volume 200 μL) and then mixed with one volume of pre-heated (37 °C) 2X Denaturing Solution, homogenized by pipetting and left on ice for 5 min. Subsequently, one volume of Acid-Phenol: Chloroform was added, and the solution was mixed thoroughly by vortexing for 60 s. The samples were then centrifuged for 5 min at 12,000× *g*. The upper aqueous phase was transferred to clean tubes and 1.25 volume of absolute ethanol was added; the solution was mixed thoroughly and loaded onto the column. After three washes with the washing solutions provided by the kit, the column was transferred into clean collection tubes and RNA was finally eluted with 25 μL of preheated (95 °C) nuclease-free water. RNA concentration was determined using a NanoDrop Spectrophotometer ND-1000 (Thermo Scientific).

### 4.10. Exosome miRNA Profiling and Real-Time PCR Analysis

Exosomal miRNAs profiling was conducted using Megaplex Pools cards A and B (Applied Biosystems, Waltham, MA, USA), which detect up to 380 different miRNAs each. An amount of 600 ng of total RNA extracted from exosomes was used for single-strand cDNA synthesis in the 7.5 μL volume. The cycling conditions were the following: 40 cycles at 16 °C for 2 min, 42 °C for 1 min and 50 °C for 1 s, followed by 85 °C for 5 min and 4 °C for 5 min. A total of 6 μL of cDNA was mixed with the TaqMan Universal PCR Master Mix and nuclease-free water in a final volume of 900 μL; 100 μL of the solution was then loaded into each port of the Array card A and B. Amplification data were analyzed using the Sequence Detector v1.7 analysis software (Applied Biosystems). 

The expression analysis of single miRNAs was performed by using the TaqMan Small RNA assay (Applied Biosystems). An amount of 10 ng of total exosomal RNA was retrotrancribed with the RT primers for miR-433 and U6 snRNA (hsa-miR-433 001028; U6 snRNA 001973; Applied Biosystems). Cycling conditions: 16 °C for 30 min and 42 °C for 30 min, followed by 85 °C for 5 min and 4 °C for 5 min. The products of retrotranscription were used to prepare real-time PCR reaction mix; qPCR miRNA primers were FAM-conjugated. Real-time PCR cycling conditions: 50 °C for 2 min and 95 °C for 10 min, followed by 40 cycles of 95 °C for 15 s and 60 °C for 60 s. Real-time PCR was conducted using the ABI Prism 7900 Sequence Detection system and the data were analyzed using the Sequence Detector v1.7 analysis software (both by Applied Biosystems).

### 4.11. SDS-PAGE and Western Blot 

Cells were lysed in RIPA buffer (1% NP-40, 0.1% SDS 50 mM Tris-HCl pH 7.4, 150 mM NaCl, 0.5% Sodium Deoxycholate, 1 mM EDTA, complete protease inhibitor cocktail and phosphatase inhibitors Na_3_VO_4_ and NaF, Sigma-Aldrich, St. Louis, MO, USA) for 20 min at 4 °C. To determine protein concentration in lysates, the Bio-Rad Protein Assay (Bio-Rad Laboratories; Hercules, CA, USA) was used according to the manufacturer’s protocol. An amount of 50 μg of total protein lysates from cells or cell-derived exosomes was separated by SDS-PAGE and blotted with transfer buffer (25 mM Tris/HCl, 20 mM glycine and 10% (*v*/*v*) methanol on nitrocellulose membranes (Whatman GmbH; Dassel, Germany). Blocking was performed with 5% milk or BSA in the TBS-0.1% Tween buffer for 1 h. Nitrocellulose membranes were then incubated with specific antibodies. Anti-mouse or anti-rabbit horseradish peroxidase-conjugated secondary antibodies and, finally, an enhanced chemiluminescence kit (Amersham, GE Healthcare; Buckinghamshire, UK), were used to detect immunoreactivity, following the manufacturer’s instructions.

Antibodies used: anti-p85, purchased from Millipore (Billerica, MA, USA); anti-phospho-p53 (Ser15) from Cell Signaling Technology (Danvers, MA, USA); anti-p53 (DO-1) from Santa Cruz Biotechnology (Santa Cruz, CA, USA); anti-p21 from Merck Millipore; anti-CDK6 (DCS83) from Cell Signaling Technology; and anti-γH2AX from Abcam (Boston, MA, USA).

### 4.12. Virus Production and In Vitro Transduction

Lentiviral vectors expressing the precursors for miR-343 (CS-HMir0214-mr03-01) or scrambled control (CS-CmiR0001-MR03-02) were purchased from Gene CopoeiaTM Rockville, MD. The virus production and infection of SKO-007(J3) cells were performed as previously described [21]. For lentiviral particle production, 293T packaging cell line at 70% confluence was transfected with miRNA expression vectors, together with the packaging vectors pVSVG and psPAX2 with Lipofectamine Plus. After an additional 48 h, the virus-containing supernatants were harvested, filtered (0.45 μm) and used immediately for infections. Infections were performed on 0.5 × 10^6^ SKO-007(J3) cells in 2 mL complete medium with polybrene (8 μg/mL) for 1 h at 37 °C. After the infection, cells were allowed to expand for 24 h and were then cultivated in medium enriched with puromycin (1 μg/mL) for negative selection. 

### 4.13. Degranulation Assay

Natural killer cell cytotoxicity was assessed by measuring the surface expression of the lysosomal marker CD107a as a surrogate for degranulation, as previously described [40]. As a source of effector cells, we used peripheral blood mononuclear cells (PBMCs) from healthy donors, isolated by Lymphoprep (Nycomed, Oslo, Norway) gradient centrifugation. After a 48 h treatment with exosomes, MM cells were incubated with total PBMCs at effector:target (E:T) ratios of 1:2.5 in complete medium. The plates were incubated at 37 °C in a 5% CO_2_ atmosphere for 2 h. Thereafter, the cells were washed with PBS and incubated with anti-CD107a/APC (or cIgG/APC) for 45 min at 4 °C. The percentage of CD107a^+^ cells was evaluated gating on CD56^+^ and CD3^−^ NK cells.

### 4.14. Statistics

Error bars represent standard deviation (SD). Statistical analyses were performed with Graphpad Prism 8.

## Figures and Tables

**Table 1:** Clinical parameters of MM patients.

N°	Sex	Age	Disease Stage	% PCs in BM
1	M	75	relapse	50
2	F	70	relapse	40
3	M	66	onset	42
4	F	63	onset	51

## Supplementary Materials

The following supporting information can be downloaded at: https://www.mdpi.com/article/10.3390/ijms24076862/s1.
