# Supplementary material for: Doxorubicin–Mediated miR–433 Expression on Exosomes Promotes Bystander Senescence in Multiple Myeloma Cells in a DDR–Independent Manner
Source: Int J Mol Sci. 2023 Apr 6;24(7):6862. doi: 10.3390/ijms24076862 (PMC10095495; doi:10.3390/ijms24076862)
Supplement: Supplementary file 1 [file ijms-24-06862-s001.zip › ijms-2283103-supplementary.pdf]

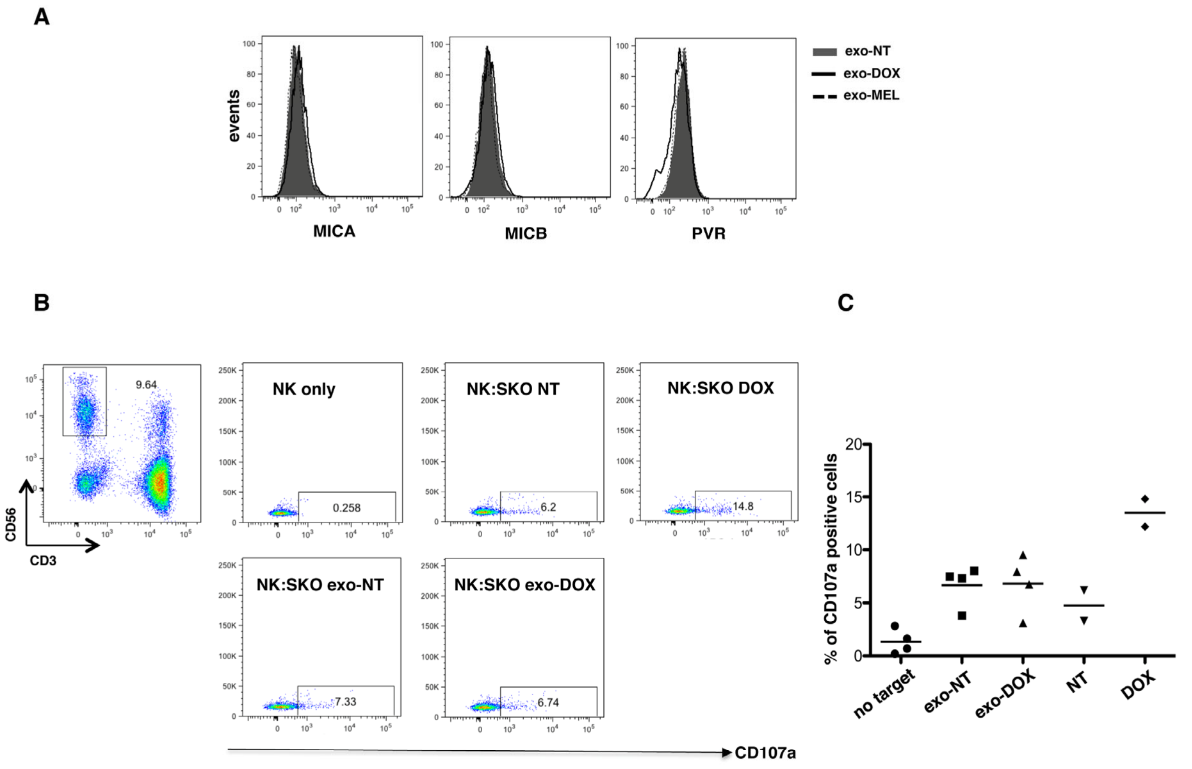

Supplemental figure 1

Figure S1. Exosomes derived from DOX-treated SKO-007(J3) cells do not affect NK cell degranulation. (A) SKO-007(J3) cells were incubated with DOX (0.05 μM) for 48 h and left for an additional 24 hours in the absence of the drug. Exosomes (50 μg/mL) were then isolated from the supernatants and used to stimulate MM cells for 48h. MICA, MICB, and PVR surface expression was analyzed by immunofluorescence and flow cytometry on SKO-007(J3) cells. Data shown are representative of one of three independent experiments. (B) Total PBMCs of healthy donors were incubated with MM cells treated for 48h with exosomes prepared as described above and used in a degranulation assay as target cells. Results are expressed as the percentage of CD107a$^+$ NK cells (gated as CD56$^+$ and CD3$^-$) obtained by subtracting the percentage of isotype control Ab and are representative of one of four independent experiments. The basal CD107a expression on NK cells was ~0.2%. (C) The average of different donors is shown as percentage of CD107a positive cells (n = 4). Doxorubicin-treated senescent MM cells were used as control.

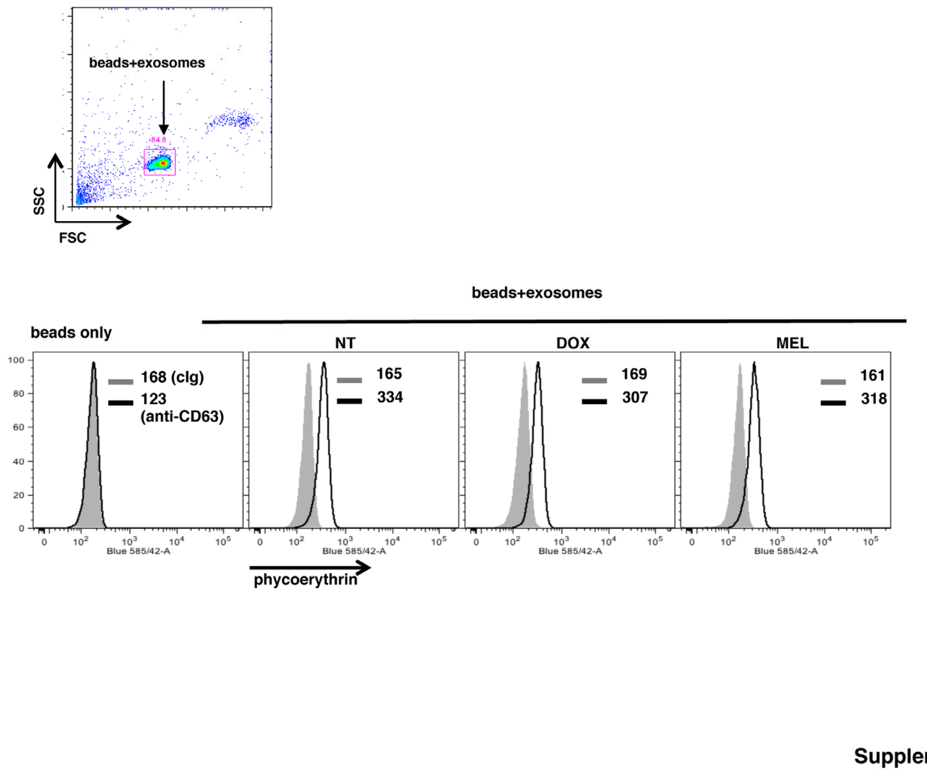

Figure S2. Exosomes derived from drug-treated SKO-007(J3) cells do not contain measurable amounts of doxorubicin.

SKO-007(J3) cells were incubated with DOX (0.05 μM) for 48 hours and left for an additional 24 hours in the absence of the drug. Exosomes were then isolated from the supernatants and incubated with CD63+ magnetic beads for 18 hours. Exosomes-beads complexes were washed and incubated with anti-CD63-PE (Phycoerythrin) or with an isotype cIg-PE. Samples were then visualized by immunofluorescence and FACS analysis. MFI (mean fluorescence intensity) are reported in each box.

	exo miR-CTR		exo miR-433	
**pp53**	1	0.85	1	0.84
**p53**	1	0.84	1	0.91
**p21**	1	0.8	1	1
**γH2AX**	1	0.9	1	1

Supplemental Table 1

Table S1. Densitometric analysis of Figure 4E.
Numbers represent densitometric analysis of different target proteins normalized to p85, used as loading control, relative to the exo-miR-CTR control treated cells.
